# Comparison between air pollution concentrations measured at the nearest monitoring station to the delivery hospital and those measured at stations nearest the residential postal code regions of pregnant women in Fukuoka

**DOI:** 10.1186/s12199-017-0663-2

**Published:** 2017-06-13

**Authors:** Takehiro Michikawa, Seiichi Morokuma, Hiroshi Nitta, Kiyoko Kato, Shin Yamazaki

**Affiliations:** 1 Environmental Epidemiology Section, Centre for Health and Environmental Risk Research, National Institute for Environmental Studies, 16-2 Onogawa, Tsukuba, Ibaraki 305-8506 Japan; 2 Department of Obstetrics and Gynaecology, Kyushu University Hospital, Kyushu University, 3-1-1 Maidashi, Higashi-ku, Fukuoka, 812-8582 Japan

**Keywords:** Air pollution, Monitoring station, Pregnant woman, Validation

## Abstract

**Background:**

Numerous earlier studies examining the association of air pollution with maternal and foetal health estimated maternal exposure to air pollutants based on the women’s residential addresses. However, residential addresses, which are personally identifiable information, are not always obtainable. Since a majority of pregnant women reside near their delivery hospitals, the concentrations of air pollutants at the respective delivery hospitals may be surrogate markers of pollutant exposure at home. We compared air pollutant concentrations measured at the nearest monitoring station to Kyushu University Hospital with those measured at the closest monitoring stations to the respective residential postal code regions of pregnant women in Fukuoka.

**Methods:**

Aggregated postal code data for the home addresses of pregnant women who delivered at Kyushu University Hospital in 2014 was obtained from Kyushu University Hospital. For each of the study’s 695 women who resided in Fukuoka Prefecture, we assigned pollutant concentrations measured at the nearest monitoring station to Kyushu University Hospital and pollutant concentrations measured at the nearest monitoring station to their respective residential postal code regions.

**Results:**

Among the 695 women, 584 (84.0%) resided in the proximity of the nearest monitoring station to hospital or one of the four other stations (as the nearest stations to their respective residential postal code region) in Fukuoka city. Pearson’s correlation for daily mean concentrations among the monitoring stations in Fukuoka city was strong for fine particulate matter (PM_2.5_), suspended particulate matter (SPM), and photochemical oxidants (Ox) (coefficients ≥0.9), but moderate for coarse particulate matter (the result of subtracting the PM_2.5_ from the SPM concentrations), nitrogen dioxide, and sulphur dioxide. Hospital-based and residence-based concentrations of PM_2.5_, SPM, and Ox were comparable.

**Conclusions:**

For PM_2.5_, SPM, and Ox, exposure estimation based on the delivery hospital is likely to approximate that based on the home of pregnant women.

**Electronic supplementary material:**

The online version of this article (doi:10.1186/s12199-017-0663-2) contains supplementary material, which is available to authorized users.

## Background

There is increasing evidence for an association between air pollution and maternal and foetal health. Since the health effects of air pollution are relatively small, a large sample size is required in order to investigate this association. Therefore, numerous earlier studies used registry data to increase the number of the participants [1–7]. In such a study design, however, personal exposure to respirable pollutants could not be measured. Thus, individual exposure to air pollutants at the participants’ home addresses has been estimated based on concentrations measured at nearby ambient air monitoring stations or on a statistical model similar to land use regression models [3–7].

Our research question is whether recent air pollution in Japan influences maternal and foetal health. In Japan, however, there is no nationwide public registry data that includes sufficient information on outcome and confounders related to maternal and foetal health. Accumulated data regarding maternal and foetal health may be available if we collaborate with obstetric health care providers. Even in this case, however, we cannot collect relevant personally identifiable information, (i.e. residential addresses) to estimate maternal exposure to air pollutants, without informed consent from the subjects [8], and it is not easy to receive such informed consent from women who have given birth and completed the obstetric follow-up process. However, we may have access to anonymised information including relevant data on pregnant women who delivered at cooperating hospitals. Although anonymised data does not allow us to estimate residence-based maternal exposure to pollutants, a majority of pregnant women reside near their delivery hospitals in Japan [9, 10]. Based on the assumption, then, that pregnant women reside near their delivery hospital, we may be able to use the air pollutant concentrations at the respective delivery hospitals as surrogate markers of pollutant exposure at their homes.

Our a priori hypothesis is that pollutant exposure estimation based on the delivery hospital approximates exposure estimation based on the home address of pregnant women. To examine this hypothesis, we used aggregated residential postal code data for pregnant women who delivered at Kyushu University Hospital and compared the air pollutant concentrations measured at the nearest monitoring station to Kyushu University Hospital with those measured at the nearest monitoring stations to the respective residential postal code regions of the women.

## Materials and methods

### Study data

Aggregated postal code data for the home addresses of pregnant women who delivered at Kyushu University Hospital in 2014 was obtained from Kyushu University Hospital, Higashi-ku, Fukuoka city, Fukuoka Prefecture. As we only collected aggregated data (e.g. the number of women who resided in the region of postal code 812-0041 was 17), we did not have individual information, such as maternal age and delivery date. In 2014, a total of 784 women gave birth in Kyushu University Hospital. Among them, we excluded 89 women who resided outside Fukuoka Prefecture because they would have returned to parental homes (according to the Japanese *satogaeri* custom) near the hospital and included in this study 695 women who resided in Fukuoka Prefecture. The study protocol was approved by the Institutional Review Board of Kyushu University, Japan, and the Japan National Institute for Environmental Studies.

### Environmental data

We used daily mean concentrations of fine particulate matter (PM_2.5_, airborne particles with a 50% cut-off level at 2.5-μm aerodynamic diameter) measured by β-ray attenuation method, suspended particulate matter (SPM, airborne particles with a 100% cut-off level at 10-μm aerodynamic diameter) measured by β-ray attenuation method, nitrogen dioxide (NO_2_) measured by colorimetry employing Saltzman reagent or chemiluminescent method using ozone, and sulphur dioxide (SO_2_) measured by conductometry or ultraviolet fluorescence method, and maximum 8-h mean concentrations of photochemical oxidants (Ox) measured by ultraviolet absorption spectrometry. These concentrations were measured at the ambient air monitoring stations representing background pollutant concentrations in the respective areas and were stored in the atmospheric environment database of the Japan National Institute for Environmental Studies. The same measurement method for each of NO_2_ and SO_2_ (chemiluminescent method for NO_2_ and ultraviolet fluorescence method for SO_2_) was used in the monitoring stations within Fukuoka city. In this study, the concentration of coarse particulate matter (coarse PM) was defined as the result of subtracting the PM_2.5_ concentration from the SPM concentration.

**Fig. 1:**
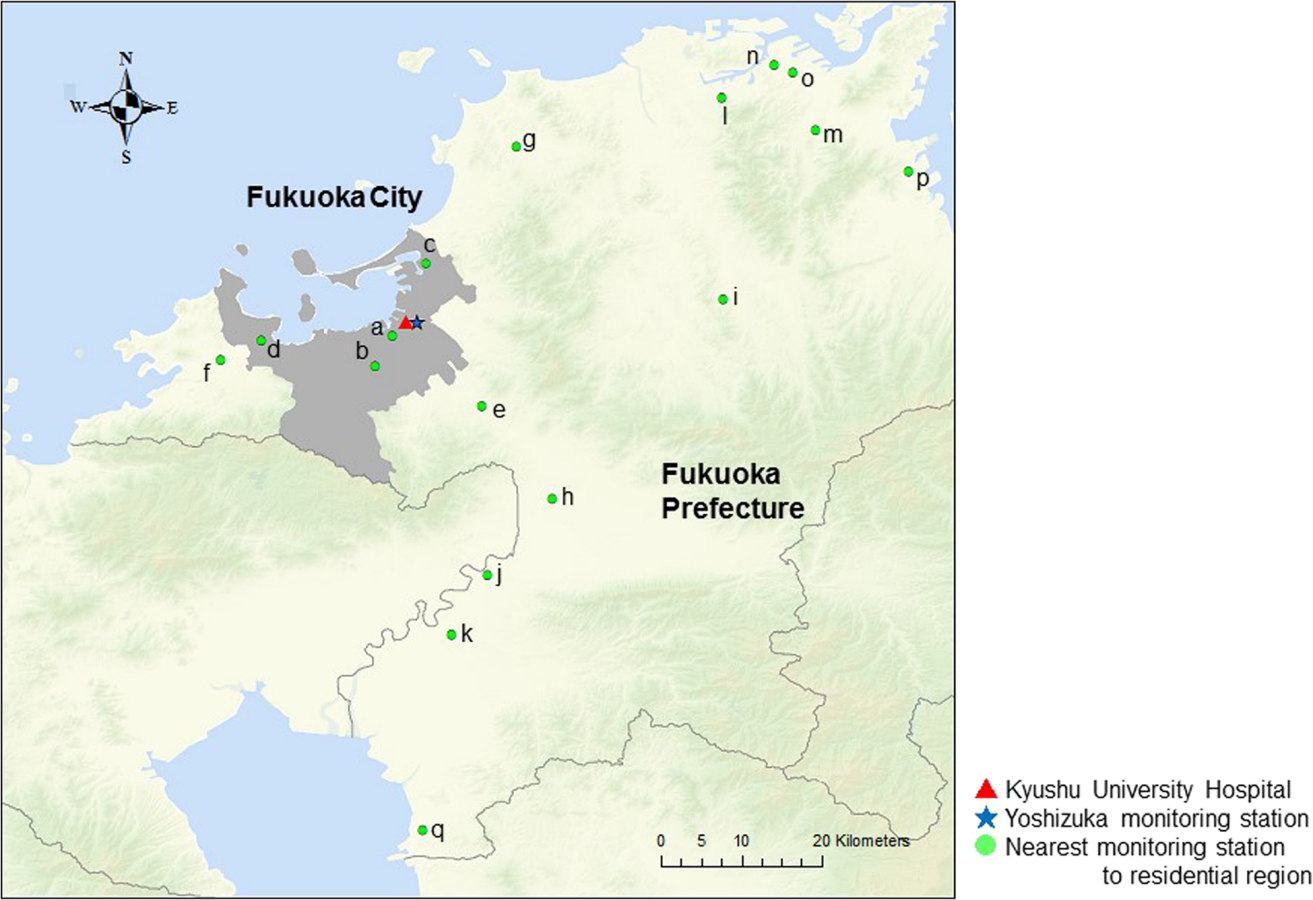
Locations of hospital and monitoring stations

We converted the aggregated data into individual data and randomly assigned, to each woman, 1 day in 2014 as a delivery day. We linked the individual delivery days and pollutant concentrations at the nearest monitoring station measuring PM_2.5_ to the respective residential postal code regions of the participants. The centroid of each region was used to measure the linear distance from the nearest monitoring station. In addition, we assigned pollutant concentrations measured at the nearest monitoring station to Kyushu University Hospital (the Yoshizuka monitoring station, approximately 1 km east of the hospital) to all participants. The hospital and monitoring station locations are shown in Fig. 1. In the past studies examining the association between air pollution and maternal and foetal health, periods of exposure estimate were set at day, week (representing a pregnancy week average), month (representing a pregnancy month average), and 3 months (representing a trimester average) [3, 7, 11–13]. Therefore, concentration values were estimated for the following periods: 1-day average (day of delivery), 7-day average, 1-month average, and 3-month average.

### Statistical analysis

We summarised residential information of study population. Pearson’s correlation coefficients of daily mean pollutant concentrations among the monitoring stations were calculated. Percentage differences were defined as the differences between concentrations measured at the nearest monitoring stations to the residential regions and those measured at the Yoshizuka station, divided by the concentrations measured at the Yoshizuka station. All analyses were performed using Stata14 for Windows (Stata Corporation, College Station, TX, USA).

## Results

Among the 695 women who resided in Fukuoka Prefecture, 641 (92.2%) resided within a 20-km radius of the Yoshizuka monitoring station, 584 (84.0%) resided near the Yoshizuka station or one of the four other monitoring stations (as the nearest stations to their respective residential postal code region) in Fukuoka city, and 279 (40.1%) resided near the Yoshizuka station (Table 1). In the case of 99.4% of the women, the linear distance between their respective residential postal code region and the nearest monitoring station was less than 10 km, while 75.7% of women resided within a 5-km radius of the monitoring station.

**Table 1:** Study population

	Number	Percent
Women who delivered at Kyushu University Hospital and resided in Fukuoka Prefecture	695	100
Women who resided within a 20-km radius of the Yoshizuka monitoring station (the nearest station to Kyushu University Hospital)	641	92.2
Women who resided near Yoshizuka station or one of the four other monitoring stations (as the nearest station to their respective residential postal code region) in Fukuoka city	584	84.0
Women who resided in Fukuoka city	433	62.3
Women who resided near Yoshizuka monitoring station (as the nearest station to their respective residential postal code region)	279	40.1

We investigated the correlation between daily mean pollutant concentrations among the monitoring stations in 2014. Among the monitoring stations in Fukuoka city (Yoshizuka and four other monitoring stations), the concentrations of PM_2.5_ were highly correlated, with Pearson’s correlation coefficients ranging from 0.96 to 0.99 (Table 2). The coefficient between PM_2.5_ concentrations measured at the Yoshizuka station and those at the farthest station from Yoshizuka station (linear distance approximately 65 km) was 0.87. Also, the concentrations of SPM and Ox were well correlated among the monitoring stations in and outside Fukuoka city (Additional file 1: Table S1). The correlation coefficients among the coarse PM concentrations measured at the Yoshizuka station and those measured at other stations ranged from 0.21 to 0.74. In the case of NO_2_ and SO_2_ concentrations, the correlation coefficients among monitoring stations in Fukuoka city, in which the same measurement method for each of NO_2_ and SO_2_ was used, ranged from 0.76 to 0.87, and the smallest coefficients between the Yoshizuka station and another station were 0.46 for NO_2_ and 0.43 for SO_2_.

**Table 2:** Pearson’s correlation coefficients for daily mean fine particulate matter (PM_2.5_) concentrations among monitoring stations in Fukuoka, 2014

Monitoring station^a^	In Fukuoka city					Outside Fukuoka city												
	Yoshizuka	a	b	c	d	e	f	g	h	i	j	k	l	m	n	o	p	q
Yoshizuka	1																	
a	0.99	1																
b	0.97	0.98	1															
c	0.97	0.97	0.97	1														
d	0.96	0.96	0.96	0.96	1													
e	0.95	0.95	0.96	0.93	0.91	1												
f	0.94	0.94	0.96	0.93	0.94	0.95	1											
g	0.94	0.94	0.94	0.94	0.92	0.95	0.95	1										
h	0.93	0.92	0.93	0.89	0.87	0.97	0.92	0.91	1									
i	0.94	0.93	0.94	0.92	0.90	0.96	0.93	0.95	0.94	1								
j	0.93	0.92	0.92	0.89	0.87	0.95	0.90	0.88	0.97	0.92	1							
k	0.82	0.79	0.78	0.75	0.72	0.82	0.75	0.72	0.88	0.78	0.93	1						
l	0.96	0.95	0.94	0.94	0.93	0.92	0.92	0.95	0.90	0.95	0.88	0.74	1					
m	0.92	0.91	0.91	0.91	0.89	0.91	0.90	0.94	0.89	0.95	0.87	0.72	0.96	1				
n	0.93	0.93	0.92	0.93	0.92	0.89	0.90	0.93	0.86	0.93	0.84	0.68	0.97	0.96	1			
o	0.92	0.92	0.92	0.92	0.91	0.90	0.90	0.93	0.87	0.94	0.85	0.70	0.97	0.97	0.98	1		
p	0.88	0.88	0.87	0.88	0.86	0.89	0.87	0.92	0.87	0.93	0.82	0.65	0.92	0.97	0.93	0.94	1	
q	0.87	0.87	0.87	0.82	0.80	0.91	0.86	0.84	0.93	0.89	0.94	0.89	0.84	0.83	0.79	0.80	0.79	1

^a^The locations of monitoring stations from a to q are shown in Fig. 1. The alphabetical order reflects the distance from the Yoshizuka station (i.e. a is the nearest station and q is the farthest station from the Yoshizuka station)

Table 3 shows comparisons between the pollutant concentrations measured at the Yoshizuka monitoring station and those measured at the nearest monitoring stations to the respective residential postal code regions. Mean PM_2.5_ concentrations at the Yoshizuka station (17.0 μg/m^3^ for 1-day average, 17.7 μg/m^3^ for 7-day average, 18.0 μg/m^3^ for 1-month average, and 18.1 μg/m^3^ for 3-month average) were similar to those at the nearest stations (17.2 μg/m^3^ for 1-day average, 17.9 μg/m^3^ for 7-day average, 18.2 μg/m^3^ for 1-month average, and 18.4 μg/m^3^ for 3-month average). Such statistical results were also observed for Ox. The percentage differences for PM_2.5_ and Ox ranged from 1.0 to 2.8%. For SPM, the percentage differences were around 6.5%, though the concentrations at the Yoshizuka station tended to be slightly lower than those at the nearest stations. The percentage differences of hospital-based and residence-based coarse PM, NO_2_, and SO_2_ concentrations ranged from 11.3 to 26.1%. Complementally, we show the correlation between pollutant concentrations measured at the Yoshizuka monitoring station and those measured at the nearest monitoring stations to the respective residential postal code regions in Additional file 1: Table S2.

**Table 3:** Comparisons between pollutant concentrations measured at the Yoshizuka monitoring station and those at the nearest monitoring stations to the respective residential postal code regions

	Concentrations measured at the nearest monitoring stations to the respective residential regions			Concentrations measured at the Yoshizuka monitoring station			Percentage difference^a^
	*n*	Mean	SD	*n*	Mean	SD	%
Fine particulate matter (PM_2.5_) (μg/m^3^)							
1-day average	689	17.2	9.2	690	17.0	9.1	1.0
7-day average	695	17.9	6.1	695	17.7	5.7	1.4
1-month average	695	18.2	4.0	695	18.0	3.5	1.2
3-month average	695	18.4	3.0	695	18.1	2.4	1.4
Suspended particulate matter (SPM) (μg/m^3^)							
1-day average	682	23.0	12.0	689	21.6	11.9	6.6
7-day average	690	24.2	8.4	695	22.7	7.9	6.7
1-month average	693	24.5	5.7	695	23.1	5.0	6.0
3-month average	695	24.7	4.8	695	23.2	3.8	6.8
Coarse particulate matter (μg/m^3^)^b^							
1-day average	676	6.0	5.2	684	4.8	5.0	24.3
7-day average	690	6.3	4.3	695	5.0	3.7	25.4
1-month average	693	6.3	3.4	695	5.1	2.5	23.2
3-month average	695	6.4	3.2	695	5.1	1.8	26.1
Nitrogen dioxide (NO_2_) (ppb)							
1-day average	685	11.6	6.7	684	14.0	7.6	−17.6
7-day average	689	11.7	5.4	695	14.0	5.3	−16.9
1-month average	692	11.8	4.8	695	14.3	4.3	−17.5
3-month average	692	11.6	4.3	695	14.4	3.7	−19.1
Photochemical oxidants (Ox) (ppb)							
1-day average	687	44.0	15.3	695	42.8	14.8	2.8
7-day average	688	44.5	11.7	695	43.4	10.9	2.5
1-month average	689	44.5	10.5	695	43.6	9.4	2.2
3-month average	693	44.3	9.1	695	43.5	7.9	1.9
Sulphur dioxide (SO_2_) (ppb)							
1-day average	387	2.1	1.6	654	1.9	1.4	12.9
7-day average	401	2.2	1.2	678	1.9	0.8	15.3
1-month average	408	2.3	1.1	695	2.0	0.7	14.2
3-month average	408	2.4	1.0	695	2.2	0.7	11.3

*SD* standard deviation

^a^(Concentrations measured at the nearest monitoring stations to the respective residential regions − concentrations measured at the Yoshizuka station) ✕ 100/concentrations measured at the Yoshizuka station

^b^Concentrations of coarse particulate matter were defined as the result of subtracting the PM_2.5_ from the SPM concentrations

## Discussion

We used the residential postal codes of pregnant women who delivered at Kyushu University Hospital and compared the pollutant concentrations measured at the nearest monitoring station to Kyushu University Hospital with those measured at the nearest monitoring stations to the respective residential regions. Hospital-based and residence-based concentrations of PM_2.5_ and Ox were similar, and the percentage differences of hospital-based and residence-based SPM concentrations were around 6.5%.

As expected, most pregnant women lived a short distance from their delivery hospital, with 40% of women residing near the Yoshizuka monitoring station, which was the nearest station to Kyushu University Hospital in Fukuoka city, and 84% of women being assigned pollutant concentrations measured either at the Yoshizuka station or at one of the other four monitoring stations in Fukuoka city. Among women aged 15–49 years who resided in Fukuoka Prefecture, roughly 35% resided in Fukuoka city [14], a relatively low proportion. Since Kyushu University Hospital is an advanced treatment hospital, the proportion of women with high-risk pregnancies delivering at Kyushu University Hospital is likely to be higher than in the local obstetric health care providers. Naturally, not all women with high-risk pregnancies would reside near Kyushu University Hospital. However, the majority of women who delivered at Kyushu University Hospital resided not far from the hospital. With regard to women with low-risk pregnancies in Fukuoka Prefecture, more than 90% seemed to give birth at the local obstetric health care providers within 30 min of their home after consideration of *satogaeri* case [10]. In general, the assumption that pregnant women reside near their delivery hospital is applicable.

Reflecting the strong correlation of PM_2.5_, SPM, and Ox concentrations among monitoring stations in and outside Fukuoka city, we observed that the PM_2.5_, SPM, and Ox concentrations at the Yoshizuka station were comparable to those at the nearest stations to the residential postal code regions in spite of exposure estimate periods. These results did not contradict the earlier findings that the spatial distribution of fine particles and oxidants was relatively uniform [15]. Since the spatial variation of coarse particles was larger than that of fine particles [16], the correlation coefficients for coarse PM among monitoring stations were not good. In the case of NO_2_ and SO_2_, the correlation of concentrations among monitoring stations suggested spatial variation in NO_2_ and SO_2_. In particular, the spatial heterogeneity of NO_2_, due to its multiple emission sources, is well known [17]. Since the same measurement method for each of NO_2_ and SO_2_ was used in the monitoring stations within Fukuoka city, the influence of the measurement method to the comparison between hospital-based and residence-based concentrations was likely to be small. Therefore, exposure estimations for coarse PM, NO_2_, and SO_2_ based on the delivery hospital must be more tentative than those for PM_2.5_, SPM, and Ox.

The present study has a number of limitations. We estimated pollutant exposure at each woman’s home using pollutant concentrations measured at the nearest monitoring stations to their respective residential postal code region. Although the majority of women resided within a 5-km radius of their respective monitoring station, our estimation still may not accurately reflect the pollutant concentrations at their homes due to spatial variation in pollutant concentrations within the region where a monitoring station covers [18, 19]. Furthermore, some women resided in areas far from Kyushu University Hospital, yet some of these women may have returned to parental homes nearer to this hospital, and thereby given birth at the hospital. Although we excluded women who resided outside Fukuoka Prefecture under the assumption of the *satogaeri* case, we did not know of any *satogaeri* case in the Prefecture. If we had excluded such *satogaeri* cases, the accuracy of the assumption that pregnant women resided near their delivery hospital may have been more clearly determined. Finally, we did not consider maternal time-activity pattern. There is a report in Canada that time-active pattern does not have a large impact on exposure estimation for pregnant women who spend a lot of time at home [20], though this may not be applied for the Japanese pregnant women.

In conclusion, our hypothesis that pollutant exposure estimation based on the delivery hospital approximates that based on the home address of pregnant women is likely to be acceptable for PM_2.5_, SPM, and Ox.

## Additional file

Additional file 1: Table S1. Pearsons’ correlation coefficients for daily mean pollutant concentrations among monitoring stations in Fukuoka, 2014. **Table S2.** Pearson’s correlation coefficients between pollutant concentrations measured at the Yoshizuka monitoring station and those at the nearest monitoring stations to the respective residential postal code regions. (XLSX 20 kb)

## Abbreviations

NO_2_: Nitrogen dioxide
Ox: Photochemical oxidants
PM: Particulate matter
PM_2.5_: Fine particulate matter
SO_2_: Sulphur dioxide
SPM: Suspended particulate matter
